# Increased intrinsic default-mode network activity as a compensatory mechanism in aMCI: a resting-state functional connectivity MRI study

**DOI:** 10.18632/aging.102986

**Published:** 2020-04-01

**Authors:** Jiali Liang, Yunfei Li, Hao Liu, Sisi Zhang, Meimei Wang, Yonghua Chu, Jianping Ye, Qian Xi, Xiaohu Zhao

**Affiliations:** 1 Department of Clinical Engineering, The Second Affiliated Hospital, Zhejiang University School of Medicine, Hangzhou 310009, Zhejiang, China; 2 Department of Imaging, The Fifth People’s Hospital of Shanghai, Fudan University, Shanghai 200240, China; 3 Department of Radiology, Shanghai Jiao Tong University Affiliated Sixth People’s Hospital, Shanghai Jiao Tong University School of Medicine, Shanghai 200233, China; 4 Department of Imaging, Shanghai Tongji Hospital, Tongji University School of Medicine, Tongji University, Shanghai 200065, China; 5 Department of Radiology, Shanghai East Hospital, Tongji University School of Medicine, Shanghai 200120, China

**Keywords:** fMRI, functional connectivity, aMCI, default-mode network, compensatory mechanism

## Abstract

Numerous studies have investigated the differences in the mean functional connectivity (FC) strength between amnestic mild cognitive impairment (aMCI) patients and normal subjects using resting-state functional magnetic resonance imaging. However, whether the mean FC is increased, decreased or unchanged in aMCI patients compared to normal controls remains unclear. Two factors might lead to inconsistent results: the determination of regions of interest and the reliability of the FC.

We explored differences in FC and the degree centrality (Dc) constructed by the bootstrap method, between and within networks (default-mode network (DN), frontoparietal control network (CN), dorsal attention network (AN)), and resulting from a hierarchical-clustering algorithm.

The mean FC within the DN and CN was significantly increased (*P* < 0.05, uncorrected) in patients. Significant increases (*P* < 0.05, uncorrected) in the mean FC were found in patients between DN and CN and between DN and AN. Five pairs of FC (false discovery rate corrected) and the Dc of six regions (Bonferroni corrected) displayed a significant increase in patients. Lower cognitive ability was significantly associated with a greater increase in the Dc of the left superior temporal sulcus.

Our results demonstrate that the early dysfunctions in aMCI disease are mainly compensatory impairments.

## INTRODUCTION

Alzheimer’s disease (AD), accounting for 60-80% of all dementia cases [1], is an irreversible neurodegenerative disease that causes progressive problems with memory, judgment, and orientation, among other functions. It has been reported that every 65 seconds, someone in the United States is diagnosed as AD, and the number of people age 65 and older with AD may grow to a projected 13.8 million by 2050 (https://www.alz.org). It is worse for AD patients over the age of 70 since 61% are expected to die before 80 years old (https://www.alz.org). However, as there is currently no treatment to prevent, cure, or slow the progression of AD, its early diagnosis is significantly important. At present, it is generally considered that mild cognitive impairment (MCI) could be the transitional state between normal cognitive functioning and dementia, which might progress to dementia, maintain stable cognitive status, and even reverse to normal [2–5]. Clinically, according to whether there is impaired memory function, two MCI subtypes have been proposed, amnestic MCI (aMCI) and non-amnestic MCI. aMCI, as one of the important forms of MCI, is mainly characterized by impaired memory function and a high risk of conversion to AD [2, 6]. Petersen and his colleagues found that aMCI is associated with an annual conversion rate to AD of 10–15% [6]. One study followed 1,265 subjects 6 years in a large sample longitudinal study, and found that 33.9% of the participants with aMCI at baseline progressed to AD [2]. Therefore, early diagnosis of aMCI and timely intervention are very important in clinical practice.

Resting-state functional magnetic resonance imaging (fMRI) is a type of noninvasive measure that enables the detection of intrinsic activity in the human brain in the nontask state. The identification of significant spatial or temporal patterns in brain activity is instrumental in the identification of neural substrates of cognition [7], which depend on dynamic interactions of distributed brain regions operating in large-scale networks [8]. The functional network represents a complex system as sets of discrete elements (nodes) and their mutual relationships (edges), which can be summarized in the form of a functional connection matrix [7]. Functional connectivity (FC), which examines spontaneous fluctuations in the blood oxygen level-dependent (BOLD) signal of fMRI [9], is an index that shows alterations before neuronal loss and structural atrophy in patients [10]. To date, a coincident observation in aMCI patients is that the mean FC strength of the default-mode network (DN) is impaired compared to that in normal control (NC) subjects [11–16]. However, whether the mean FC strength of the DN in aMCI patients is increased, decreased or unchanged is still controversial. Wang [11] and Binnewijzend [16] did not find any significant differences in resting state FC strength within the DN when comparing aMCI patients with NC subjects. Li [13] demonstrated that the mean FC strength of DN was reduced in aMCI patients, whereas Gardini [15] indicated that this value was higher in aMCI patients. Despite the different conditions of aMCI patients, the analysis of FC is subject to a limitation in the definition of regions of interest (ROIs) [7] and reliable edges.

Previous studies have strongly demonstrated that activity increases during rest in DN regions, which are dysfunctional in AD and aMCI. However, the regions within the DN will sometimes move to another network in different participants. For example, the precuneus (PCu), belonging to the DN in one study [17], drifted to the frontoparietal control network (CN) in another study [18]. Therefore, a single atlas preset for the default network cannot match all subjects, and reorganizing the ROIs into their correct affiliations is of great importance for each investigation. In the present study, a hierarchical-clustering algorithm was applied to ROIs defined by more reliable task-based activation [18, 19] rather than an anatomical parcellation. Not only DN was considered but also the CN and the dorsal attention network (AN) were taken into account, because it has been demonstrated that the DN has a close relationship with the AN and the CN [17].

Another significant cause is the spurious functional connections that unquestionably hamper our analysis. Fully connected time-courses of ROIs, without any restrictions of setting a threshold value (for example, setting a threshold correlation coefficient larger than 0.2 [13]) or choosing a connection density (for example, 20% connection density [20]), will result in undesirable noise that forms spurious functional connections. Some weak but relatively reliable FC that may play a significant role in a brain network will be lost, whatever threshold value or connection density is selected. In fact, one region predominantly interacts only with a small number of regions [21]. To construct a more reliable human brain network model, a method should be introduced to hold these weak but reliable FC. In the present study, a bootstrap method was applied to the fMRI data of aMCI and normal controls. The bootstrap method, introduced by Efron in 1979 [22], is a sampling-based approach which is used to generate a huge number of bootstrap samples though randomly resampling with replacement. The statistic of interest will be estimated according to these bootstrap samples. It has an advantage of a relatively high accuracy of the parameter estimation [23], especially for those small size samples.

In addition to the focus on the FC between regions, the node metric, degree centrality (Dc), was also observed. Dc quantifies the importance or centrality of a node through the strength of connections to all of the other nodes in a weighted network. It has been adopted over other nodal centrality approaches because it has been proven to be more robust [24, 25].

In this study, for the first time, we explored the difference in FC and Dc constructed by the bootstrap method, between and within networks resulting from a hierarchical-clustering algorithm for the aMCI and normal subjects. In addition, we also investigated the relationship between the Mini Mental State Examination (MMSE) scores and FC as well as Dc.

## RESULTS

**Figure 1:**
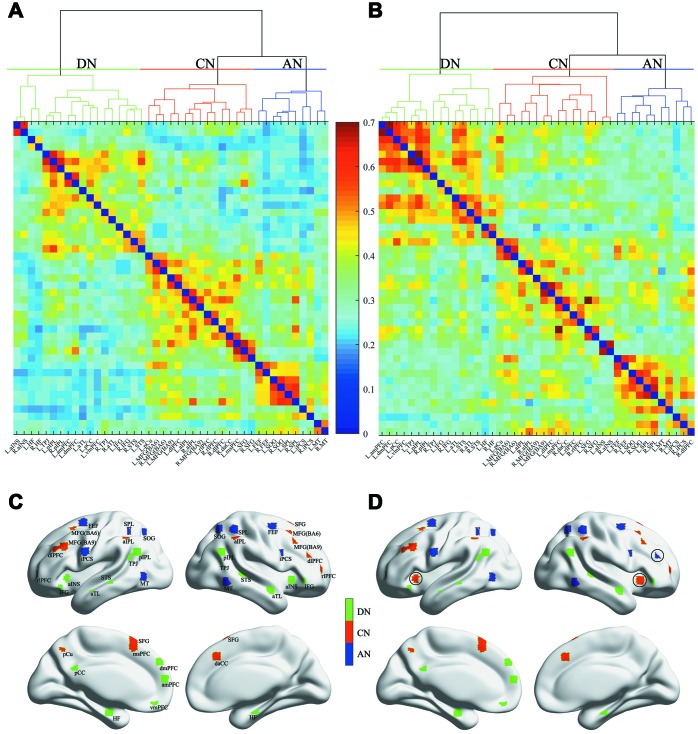
**Dendrogram of the hierarchical cluster analysis of the correlations and spatial distribution of the three networks.** (**A**) and (**B**) separately represent the NC group and aMCI group, and the colors indicate the magnitude of correlation. (**C**) and (**D**) separately represent the NC group and aMCI group. The green, red and blue regions indicate the DN, CN and AN, respectively.

**Figure 2:**
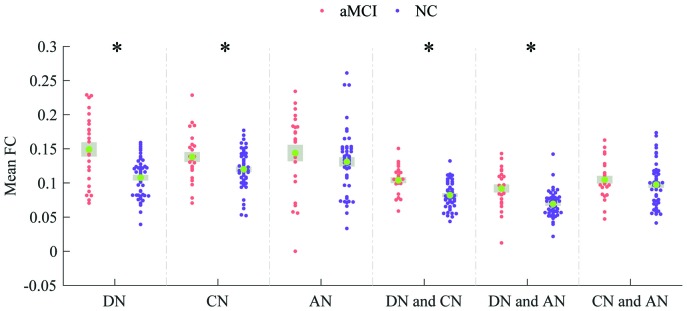
**Comparisons of mean FC within- and internetwork.** Patients and the normal persons are colored in Indian red and dark orchid, respectively. * *P* < 0.05 (uncorrected).

**Figure 3:**
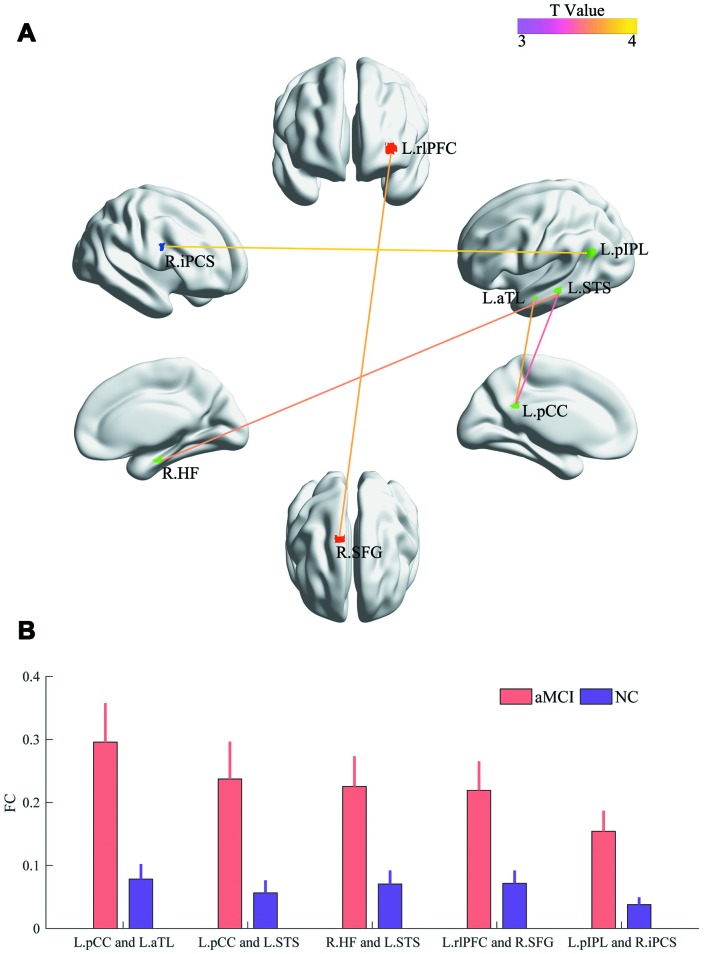
**Comparisons of pairwise FC.** (**A**) The colored line indicates the T values. (**B**) The bar graph displays statistical differences (FDR corrected) in FC between the two groups.

The three networks (DN, CN and AN) of both NC subjects and aMCI patients are shown in Figure 1A and 1B, respectively. Figure 1C and 1D separately show the spatial distributions of the normal and patient groups. The green, red and blue regions express the DN, CN and AN, respectively. The number of overlapped regions between the networks obtained by the hierarchical-clustering algorithm and the task-defined networks were that 14 (14/16) for DN, 12 (12/14) for CN, and 13 (13/13) for AN in the normal group, and 16 (16/16) for DN, 12 (12/14) for CN, and 12 (12/13) for AN in the patient group, respectively. The graphs were visualized with the BrainNet Viewer (http://www.nitrc.org/project/bnv/) [26]. Compared with the normal group, the patient group revealed that 3 regions (bilateral anterior insula (aINS) and the right dorsolateral prefrontal cortex (dlPFC)) shifted in their network affiliation and are marked with black circles in Figure 1D. Bilateral aINS shifted from DN to CN, and the right dlPFC belonging to CN transferred to AN. The mean FC values within the DN (*P* = 2.82 × 10^-5^) and CN (*P* = 2.98 × 10^-2^) showed significant increases in the patient group (Figure 2). For the internetwork pairs, significant changes were found in DN between CN (*P* = 5.31 × 10^-5^) and DN between AN (*P* = 1.97 × 10^-4^) (Figure 2). The statistical comparison of pairwise FC is shown in Figure 3. In Figure 3A, the colored line indicates the T value, and the bar graph displays the statistical differences (false discovery rate (FDR) corrected) in FC between the two groups.

**Figure 4:**
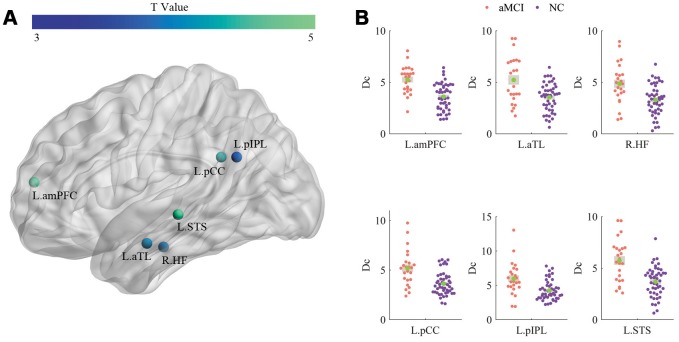
**Comparisons of Dc.** (**A**) and (**B**) illustrate the T values and significant alterations (Bonferroni corrected) between the two groups, respectively.

The Dc values of the 6 regions were significantly increased in the patient group, and panels (A) and (B) of Figure 4 illustrate the T values and the significant alterations (Bonferroni corrected) between the two groups, respectively.

**Figure 5:**
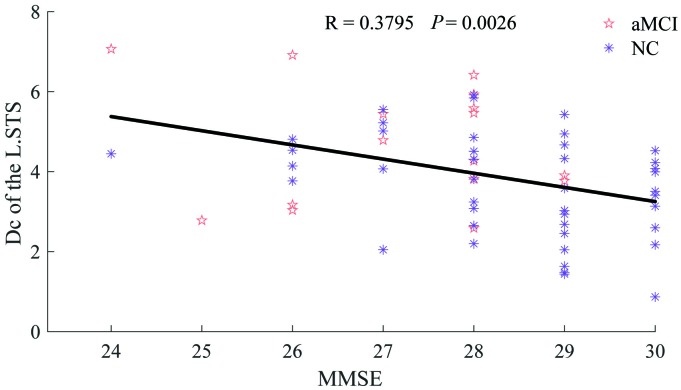
**Relationship between Dc of the L.STS and MMSE.** The patient group is indicated by the Indian red pentagrams and the normal group by dark orchid asterisks.

The Dc of the left superior temporal sulcus (STS) was significantly (R = 0.3795, *P* = 0.0026) correlated with MMSE, as shown in Figure 5. Lower cognitive ability (lower MMSE scores) was significantly associated with a greater increase in the Dc of the left STS. No significant relationship between FC and MMSE was detected.

## DISCUSSION

The mean FC strengths of the default and control networks were significantly increased in the aMCI patients and significantly grew between the DN and AN, DN and CN for the patients. Further research suggested that five stronger pairs of FC strength were discovered in the patient group (left posterior cingulated cortex (L.pCC) and left anterior temporal lobe (L.aTL), L.pCC and L.STS, right hippocampal formation (R.HF) and L.STS, left rostrolateral prefrontal cortex (L.rlPFC) and right superior frontal gyrus (R.SFG), left posterior inferior parietal lobule (L.pIPL) and right inferior precentral sulcus (R.iPCS)). Meanwhile, six regions (left anterior medial prefrontal cortex (L.amPFC), L.aTL, R.HF, L.pCC, L.pIPL, L.STS) located in the default network showed significantly higher Dc for the patients, and one of the regions (L.STS) displayed a significantly negative relationship with MMSE.

### Methodological improvements

The hierarchical-clustering algorithm might be an optional way of classifying brain networks. It might be inappropriate to classify regions into different networks only according to previous research, neglecting the characteristics of a study’s own samples, such as age and health. For example, middle frontal gyrus (MFG) was defined as one region of DN across three subject groups (young controls, elderly controls and AD patients) [27], whereas MFG was determined to be one of the regions of CN during every condition (i.e., rest state and task state) [17]. A possible approach is to reorganize brain regions by utilizing the hierarchical-clustering algorithm. Meanwhile, it has been demonstrated that the hierarchical clustering method has a high accuracy for the analysis FC patterns [28]. The results obtained by these means were essentially in line with previous studies [17, 18, 29].

Spurious functional connections existing in fully connected correlation coefficient matrices must be removed to obtain a more realistic model. However, most scientists construct these matrices without any filtration. Of note, we also compared the two groups, as most researchers, but no significant differences (*P* = 0.05, uncorrected) were found. Clearly, investigators are eager to discover even a slight difference between early patients and normal persons for the purpose of early diagnosis and treatment. The bootstrap method might be a feasible approach for exploring slight changes since it could retain weak but reliable functional connections. In the current study, for the first time, we adopted a bootstrap resampling procedure to dispose the fMRI data of normal subjects and aMCI patients from the aspect of emphasizing the reliability of FC. Interesting, the significant difference (*P* < 0.05, FDR corrected) in FC with relatively low magnitude (mean FC < 0.2, as shown in Figure 3) of a single pair (left pIPL and right iPCS) was detected. The left pIPL and the right iPCS belonged to the default network and the attention network, respectively. The appearance of a change in FC between these two regions should be given attention because the relationship between the default network and the attention network is modulated by the control network in healthy persons, and is not correlated with each other directly, according to a previous study [17]. The abnormal findings in FC between DN and AN in aMCI patients might be an early neuroimaging characteristic. However, this hypothesis needs more evidence to be proven.

### The functions of DN and its correlations with memory impairments in aMCI

The main clinical feature of aMCI and AD patients is the impairment in the functions of cognition and memory, and these functions have a close relationship with the activity of the DN. To date, the impairment of the DN in aMCI patients has been confirmed, but the nature of this change is still under debate. Compared with the mean FC of the DN in normal persons, the value in MCI patients was increased [15], decreased [13, 14], or unchanged [11]. These inconsistencies [11, 14] might derive from the spurious FC. Even though Li [13] set a threshold value for the correlation coefficient to eliminate the noise, the different stages of patients should be noted, according to the findings of Tao [30]. Judging only from the MMSE values in Table 1 of this article [30], our patients could roughly be determined to have mild aMCI, whereas their patients [13] had severe aMCI. Different variations in the mean FC of the default network for different stages of MCI patients should be addressed carefully.

**Table 1:** Demographics and clinical data of the aMCI patients and the NC group.

	**aMCI**	**NC**	***P* value**
Age (year)	73.60 ± 7.26	70.67 ± 7.00	0.099^a^
Education (year)	12.28 ± 3.12	12.57 ± 3.08	0.707^a^
Gender (M/F)	17/8	26/25	0.160^b^
MMSE	27.52 ± 1.44	28.33 ± 1.34	0.019^a^
Head motion	0.12 ± 0.07	0.11 ± 0.07	0.697^a^

Data: mean ± standard deviation (SD)

^a^The *P* value was obtained by a two-sample two-tailed *t* test

^b^The *P* value was obtained by a two-tailed Pearson chi-square test

The higher mean FC of the default network in aMCI patients may reflect a compensatory mechanism. This mechanism often assumes that overactive regions are “working harder” to make up for functional declines elsewhere in the brain [31]. For example, the increased betweenness centrality of the left lingual gyrus represented a compensatory process for the reduced centrality in other regions [32]. However, the decreased FC of the default network in patients was not found in our results. On one hand, we speculated that the increased FC in patients at rest might be used to compensate the decreased FC during task. Of note, a higher mean FC of the default network in the rest state does not indicate better functions, such as episodic memory and autobiographical memory, during tasks. In contrast, it has been proven that higher FC is associated with poorer cognitive performance [33], and stronger compensation coupled with impairments suggests that a patient with worse MCI progresses to AD [34]. On the other hand, we hypothesized that this compensatory mechanism could alternatively be interpreted as the diminished ability of functional connectivity to decline. For normal controls, their great levels of deactivation, particularly in the default network during a nontask period, are associated with massive cognitive effort during tasks [35]. However, for aMCI patients, as the ability of FC to decline has decreased, the FC strength of patients is higher than that of normal controls, and the demanded cognitive energy is inadequate during tasks, ultimately resulting in poorer cognitive performance.

### Conceptual meaning of Dc and its relationship with memory impairments in aMCI

The Dc of a node is determined by the number and strength of functional connections and is adopted to indicate the relevance of this node for the information flow in the brain network [36, 37]. The greater the Dc, the more important it is. Higher Dc was found in patients, as shown in Figure 4, and the Dc of the left STS was inversely correlated with MMSE, as presented in Figure 5. The reason for the increased Dc of the DN in patients was probably to partially reorganize the brain for the adaptation of neurodegeneration. Furthermore, the high level of metabolism in the default network is conducive to the formation of pathology associated with AD [38].

### Abnormal modulation between networks

Early studies exploring MCI patients usually focused their attention on a single network, i.e., the default network, but we should always consider that functional connectivity works across multiple networks. This viewpoint has been proven in recent years, and the three networks (DN, CN, AN) are closely related to patients with MCI [39–41]. The CN also presented a higher mean FC in patients, possibly because the CN might flexibly mediate the default network in support of intrinsic activity during the resting state [18]. The mean FC of AN did not differ between the two groups, probably because of the strong anticorrelated and competitive relationship between DN and AN [38, 42].

Increases in mean FC between DN and CN and between DN and AN were detected in the patients, while there was no difference in the mean FC between CN and AN, as seen in Figure 2. The impairment between networks revealed that the default network plays a critical role as a bridge linking the other two networks.

### Limitation

There are 3 issues in need of improvement. First, the sample problem. It is generally known that aMCI subjects are divided into mild, moderate, and severe stages. Finer classifications of patients would be very beneficial for us to comprehensively understand this disease. Second, the faultiness of network selection. As more and more studies have demonstrated that neurological disease is associated with not only the three networks we investigated but also other networks, such as the salience network and the sensorimotor network, more related networks should be involved in future studies. Third, a defect in the bootstrapping procedure. This method overemphasizes the reliability of the FC, but overlooks the number of edges. Specifically, a different number of edges could confound between-group comparisons [43].

## CONCLUSIONS

The novel finding of the present study is that for the first time, we explored the difference in FC and Dc constructed by the bootstrap method, between and within networks, resulting from a hierarchical-clustering algorithm for aMCI and normal subjects. Increases in mean FC within and between networks, FC between regions, and Dc were discovered in aMCI patients, indicating the early dysfunctions of this illness and this approach opens the doors for investigations into other brain diseases.

## MATERIALS AND METHODS

### Subjects

Eighty-four volunteers were recruited in this study, including 27 aMCI and 57 NC subjects. The neurologic examination and neuropsychological measurements completed by neurologist and certified study psychometrists from Institute of Neurology, Huashan Hospital, Fudan University, Shanghai, China [44]. The criteria are based on the diagnostic criteria proposed by Petersen [45]: (1) memory complaints usually corroborated by an informant; (2) objective memory impairment for age; (3) essentially preserved general cognitive function; (4) largely intact functional activities; and (5) not demented [46]. The exclusion criteria were as follows: (1) a history of neurological or psychiatric or head injury; (2) current treatment with vasoactive or psychotropic medication; (3) any physical or intellectual disability; (4) any contraindication to MRI. All participants provided written consent. Ultimately, 25 aMCI and 51 NC persons were selected for the research study after excluding 8 subjects due to excessive head motion (see data preprocessing). Table 1 shows the details of the clinical and demographic data of the remaining subjects in the two groups.

### Data acquisition

The MRI scans were performed at the Shanghai Tongji Hospital, China, with a Siemens scanner. Foam padding and headphones were used to limit head motion and reduce scanner noise. fMRI data were acquired using echo planar imaging (EPI) with repetition time (TR) = 2 s, echo time (TE) = 30 ms, flip angle (FA) = 90 °, matrix = 64 × 64, voxel size = 3.44 × 3.44 × 4.29 mm^3^, number of slices = 31, and slice thickness = 3.8 mm. T1-weighted anatomical images were scanned with the following parameters: TR = 2.53 s, TE = 2.34 ms, FA = 7 °, inversion time (TI) = 1.1 s, and slice thickness = 1 mm. During the fMRI scans, subjects were instructed to hold still, remain motionless, and think of nothing in particular.

### Data preprocessing

All images were preprocessed using the Statistical Parametric Mapping (SPM12, http://www.fil.ion.ucl.ac.uk/spm/) software package and Data Processing and Analysis for (Resting-State) Brain Image (DPABI) [47]. The first five time points were discarded for signal equilibrium and the participants’ adaptation to the MRI scanning. The images were then slice time-corrected and realigned. Two aMCI patients and six NC persons were excluded due to excess head motion (rotation > 2 ° or translation > 2 mm [48]). T1-weighted images were subsequently coregistered to the mean functional images, followed by segmentation into gray matter (GM), white matter (WM) and cerebrospinal fluid (CSF) [49]. Each bad time point, defined as volumes with framewise displacement (FD) (Jenkinson) > 0.2 mm, as well as volumes 2 forward and 1 back from these volumes, was included as a regressor [47, 50], after regressing out head motion effects from the realigned data using the Friston 24-parameter model [51]. Additionally, WM and CSF signals were regressed out to reduce respiratory and cardiac effects, whereas the global signal was not regressed out due to the controversy that its removal would cause redistribution of correlation coefficients [52]. After that, temporal filtering (0.01 – 0.08 Hz) was applied to the time series to decrease the effects of low-frequency drifts and high-frequency physiological noise. The functional volumes were spatially normalized to the Montreal Neurological Institute (MNI) space and resampled to 3-mm isotropic voxels. Finally, the functional images were spatially smoothed with a Gaussian kernel of 6 × 6 × 6 mm^3^ full width at half maximum (FWHM).

### Determining ROIs

Forty-three ROIs defined by task-based activation in previous studies [18, 19] were selected in this research, and the radius of each region was 3 mm. Forty-three ROIs representing the core nodes of these three networks were derived from a previous study [17] in which the ROIs were isolated by a multivariate spatio-temporal analysis of three tasks: autobiographical planning, visuospatial planning, and counting. In general, the autobiographical planning task involved internally directed cognition and engaged the default mode network, the visuospatial planning task involved externally directed cognition and engaged the dorsal attention network, and both planning tasks engaged the frontoparietal control network relative to counting task. It has been demonstrated that these task-defined networks showed similar topographical patterns to intrinsic connectivity networks identified using resting-state fMRI [18, 19]. All nodes, anatomical labels and their abbreviations are listed in Table 2. The mean time series was estimated by averaging the time series of all voxels in their own regions. The FC matrices, also called correlation matrices, were obtained by calculating Pearson correlation coefficients of time series. In this study, we’d like to study the absolute variation in the FC and thus used the absolute values of both positive and negative correlations for the group comparison. Subsequently a mean correlation matrix was generated by averaging the correlation coefficient matrices of all participants in each group separately. Two mean correlation matrices, one for the aMCI group, and the other one for the normal group, were separately produced. Last, a hierarchical-clustering algorithm was separately applied to the two mean correlation matrices and divided the forty-three regions into three networks. The network was determined through visual inspections by the experienced radiologists. For a more precise definition of each network, we further explored the number of overlapped regions between the networks obtained by the hierarchical-clustering algorithm and the task-defined networks.

**Table 2:** Anatomical regions and their abbreviations.

**Region**	**Abbrev.**	**Region**	**Abbrev.**
Anterior medial prefrontal cortex	amPFC	Anterior temporal lobe	aTL
Dorsal medial prefrontal cortex	dmPFC	Hippocampal formation	HF
Inferior frontal gyrus	IFG	Posterior cingulated cortex	pCC
Posterior inferior parietal lobule	pIPL	Precuneus	PCu
Superior frontal gyrus	SFG	Superior temporal sulcus	STS
Temporal parietal junction	TPJ	Frontal eye fields	FEF
Ventral medial prefrontal cortex	vmPFC	Inferior precentral sulcus	iPCS
Middle temporal motion complex	MT	Superior occipital gyrus	SOG
Superior parietal lobule	SPL	Anterior insula	aINS
Anterior inferior parietal lobule	aIPL	Dorsolateral prefrontal cortex	dlPFC
Dorsal anterior cingulated cortex	daCC	Middle frontal gyrus BA6	MFG(BA6)
Medial superior prefrontal cortex	msPFC	Middle frontal gyrus BA9	MFG(BA9)
Rostrolateral prefrontal cortex	rlPFC		

### Constructing FC and calculating Dc

The reliable correlations were determined by implementing a bootstrapping procedure, similar to a previous study [17]. Resample over the correlation coefficients, constructing bootstrap samples. A bias-corrected and accelerated percentile method was used to determine the 95% CI for each pair of correlation. A resampling rate of 10,000 was selected to ensure the reliability and stability of each CI estimate. The bias-corrected and accelerated percentile method (BCa) is a kind of way of estimating confidence intervals of the parameter of interest, according to the bootstrap samples created though resampling with replacement. It has a relatively high accuracy of the parameter estimation [23], especially for the evaluation of a small size sample. Finally, the correlation coefficient value was preserved if it was located within the 95% CI of the mean functional connectivity strength, or was set to zero if not. Five persons (four NC subjects and one aMCI patient) were discarded, because all their correlation coefficients were outside of the 95% CI, resulting in five 43*43 zero matrices. In total, 24 patients and 47 normal subjects remained. A Fisher’s transformation was utilized to improve the normality of the correlation coefficients for every subject. The nodal parameter Dc was computed via a GRaph thEoreTical Network Analysis (GRETNA) toolbox [53].

### Statistical analysis

The statistical differences in the mean FC between and within networks were assessed using a two-sample two-tailed *t* test, with a statistical significance level of *P* < 0.05 (uncorrected). For the multiple comparisons in FC, a FDR corrected at a *q* value of 0.05 was utilized, while the multiple comparisons in Dc, a Bonferroni corrected was used. Multiple linear regression analyses were conducted to remove the confounding effects of age, gender, education, and relative Root-Mean Squared-FD.

The relationships between MMSE scores and FC as well as Dc were also investigated via a correlation analysis, with a statistical significance level of *P* < 0.05 (uncorrected) because these relationships were exploratory in nature.
